# Losing bigly

**DOI:** 10.15252/embr.202256224

**Published:** 2022-10-12

**Authors:** Howy Jacobs

**Affiliations:** ^1^ Tampere University Tampere Finland; ^2^ La Trobe University Melbourne Vic Australia

**Keywords:** Economics, Law & Politics, History & Philosophy of Science

## Abstract

What are the effects of the trend towards ever larger academic units and can we resist it

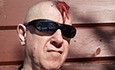

It has become a frequent occurrence in academia that institutes, departments, faculties, and whole universities periodically change their name, their logo, their corporate image, and so on. Sometimes, it is for sound reasons, such as the threat of legal action by another entity whose name they appear to have plagiarized. In other cases, a change of identity is simply a mark of a failing institution—a fresh start under a new name that carries no baggage and may also sound grander and trendier than what preceded it.

But name changes are more often associated with broader reorganizations based on the recommendations of management consultants, in which increasing size is an almost universal trend. The motivation or impetus for such amalgamations rarely comes from the scientists and teachers working in the organization. Instead, it is imposed in a top‐down fashion, with one or both of two justifications tagged on: larger units mean economies of scale because all the services that were previously duplicated can now be merged into much more efficient versions to serve a wider community of like‐minded souls. Alternatively, the academic version of trickle‐down economics is applied, though rarely is this made explicit. Typically, a successful department or institute is fused with one or more that has enjoyed less success, with the expectation that good practice or scientific brilliance of the one will inspire the other to perform better. Moreover, since academic prowess usually goes hand in hand with financial solvency based on winning external grants and the overheads that flow in their wake, the improved academic performance of the combined institute will help cover a financial hole that further tarnished the reputation of the weaker component.

In my own career, I have seen this process play out numerous times, both in the faraway lands of colleagues and collaborators as well as closer to home. And the end result is rarely the blissful, harmonious, scientific, and financial win‐win that was envisaged. This may be for many reasons but most obviously because the above assumptions are wrong. And, on top of that, larger units of organization carry their own invisible cost.

The assumption that services are delivered poorly because they are organized on too small a scale invariably hides the fact that they are already on too small a scale for the units that they serve. Effectively making the same services supply a larger unit imposes on them even greater stress with a high probability of failure. It leads to rapid staff turnover, with positions filled by new trainees to replace the experienced experts who used to run the show, and who were known and respected by those whom they served.

Fusions and amalgamations do sometimes break down unnecessary boundaries between academics working in separate but similar departments. However, this bland statement ignores the way we work, at least nowadays. Research is typically undertaken by a team led by one or sometimes two PIs. Such groups vary in size but operate best when everyone knows each other, the team has a good mix of complementary expertise, and juniors are mentored by more experienced scientists. The optimal size varies according to discipline and topic but in molecular biology seems to be somewhere between 7 and 15 persons, in my experience. This does not prevent the PI or any other member of the team from interacting with other teams in the institute or elsewhere. What does impede such interactions, apart from personal issues that can arise in any workplace, is not that the research unit as a whole is too small—specific expertise can always be found on the outside. In fact, the opposite is true: in a very large unit, people do not know one another, thus are unaware of what skills or expertise are available, are shy to approach people they do not know, and overall interactions end up less than in a small unit. The classic coffee‐room conversations out of which many valuable ideas arise are missing or muted. Result: less productivity, less workplace satisfaction, less creative thinking, and loss of a sense of identity.

Another reason why larger and larger units can be self‐defeating is that if basic services end up being delivered less efficiently, rather than more, scientists at every level have to fill in the gaps themselves. We are all stressed already by the increasing number of mundane and seemingly pointless administrative tasks to fulfill, which were previously done by a well‐trained and efficient staff who actually seemed to enjoy them. And we end up having less time for science: not just for pipetting at the bench or running an analysis program, but for thinking, which is supposedly what we were educated for.

In my experience, university teaching operates similarly. A small cluster of teachers, who know each other and deliver a well‐defined course that all have had a hand in designing, is much more effective than a large amorphous group of teachers delivering a complex set of overlapping modules, where duplications, gaps, and unevenness abound. Breaking down the boundaries just leads to a loss of coherence and unhappy students.

The trickle‐down concept also fails. Again, it ignores who we are and how we work. Wherever we are in the scientific realm, we all strive to be as inventive, as creative, and as ingenious as possible, and to be recognized for the best science we can conduct. But lumping us together with an inappropriate cohort of colleagues invariably harms both. Those who have enjoyed great success suddenly find themselves combined with “losers,” and their previous A* department is now at best a B minus. Those on the other side, who feel that they were doing their best, and most likely feeling the pressure of an unfair lack of recognition, now have to cope with the resentment of snobbish colleagues who treat them like “losers.” The result is a universal sense of grievance. Rather than trickle‐down, I would describe the outcome as Pharaoh's dream—the thin cows devouring the fat ones yet getting no fatter.

The widespread failure of larger units to perform better should logically provoke a rethink as to whether the underlying assumptions were valid. Unfortunately, the trend that most of us experience is that the management consultants behind the reorganizations foisted upon us interpret their failure as proof that bolder steps are needed, where even larger entities must be created.

But the root of the problem may not lie in those consultancies, whose recommendations might, conceivably, be appropriate for, say, a paperclip manufacturer. The fault lies with university and institute management that blithely assume that research and education are equivalent to paperclip production. Presumably, this is because they have themselves been recruited from the paperclip industry or have been away from the bench or the lectern for so many years that they have simply forgotten what science and university‐level teaching is all about.

Nevertheless, the trend towards larger and larger units, with less and less human contact, a lack of *esprit de corps*, and ever‐decreasing productivity plagues many organizations, not just academia. But perhaps we, as society's conscience, should be the first to rebel against this pointless and debilitating trend, putting our own houses in order as a first step.

